# Long-term health outcomes and cost-effectiveness of a computer-tailored physical activity intervention among people aged over fifty: modelling the results of a randomized controlled trial

**DOI:** 10.1186/1471-2458-14-1099

**Published:** 2014-10-23

**Authors:** Denise A Peels, Rudolf R Hoogenveen, Talitha L Feenstra, Rianne HJ Golsteijn, Catherine Bolman, Aart N Mudde, Gerrie CW Wendel-Vos, Hein de Vries, Lilian Lechner

**Affiliations:** Department of Psychology and Educational Sciences, Open University of the Netherlands, PO Box 2960, 6401 DL Heerlen, The Netherlands; Centre for Nutrition, Prevention and Health Services, National Institute for Public Health and the Environment (RIVM), Bilthoven, The Netherlands; Department of Health Promotion, Maastricht University, Maastricht, The Netherlands; Caphri School of Public Health and Primary Care, Maastricht University, Maastricht, The Netherlands; University Medical Centre Groningen, Groningen, The Netherlands

**Keywords:** Cost-effectiveness, Modelling, Quality of life, Disease incidence, Physical activity, Tailored intervention, Print-delivered, Web-based

## Abstract

**Background:**

Physical inactivity is a significant predictor of several chronic diseases, becoming more prevalent as people age. Since the aging population increases demands on healthcare budgets, effectively stimulating physical activity (PA) against acceptable costs is of major relevance. This study provides insight into long-term health outcomes and cost-effectiveness of a tailored PA intervention among adults aged over fifty.

**Methods:**

Intervention participants (*N* = 1729) received tailored advice three times within four months, targeting the psychosocial determinants of PA. The intervention was delivered in different conditions (i.e. print-delivered versus Web-based, and with or without additional information on local PA opportunities). In a clustered RCT, the effects of the different intervention conditions were compared to each other and to a control group. Effects on weekly Metabolic Equivalents (MET)-hours of PA obtained one year after the intervention started were extrapolated to long-term outcomes (5-year, 10-year and lifetime horizons) in terms of health effects and quality-adjusted life years (QALYs) and its effect on healthcare costs, using a computer simulation model. Combining the model outcomes with intervention cost estimates, this study provides insight into the long-term cost-effectiveness of the intervention. Incremental cost-effectiveness ratios (ICERs) were calculated.

**Results:**

For all extrapolated time horizons, the printed and the Web-based intervention resulted in decreased incidence numbers for diabetes, colon cancer, breast cancer, acute myocardial infarctions, and stroke and increased QALYs as a result of increased PA. Considering a societal Willingness-to-Pay of €20,000/QALY, on a lifetime horizon the printed (ICER = €7,500/QALY) as well as the Web-based interventions (ICER = €10,100/QALY) were cost-effective. On a 5-year time horizon, the Web-based intervention was preferred over the printed intervention. On a 10-year and lifetime horizon, the printed intervention was the preferred intervention condition, since the monetary savings of the Web-based intervention did no longer outweigh its lower effects. Adding environmental information resulted in a lower cost-effectiveness.

**Conclusion:**

A tailored PA intervention in a printed delivery mode, without environmental information, has the most potential for being cost-effective in adults aged over 50.

**Trial registration:**

The current study was registered at the Dutch Trial Register (NTR2297; April 26th 2010).

**Electronic supplementary material:**

The online version of this article (doi:10.1186/1471-2458-14-1099) contains supplementary material, which is available to authorized users.

## Background

Physical inactivity is identified as the fourth leading risk factor for global mortality and a significant predictor of several chronic diseases [1–3]. The adverse health effects of insufficient physical activity (PA) result in high costs to society [4]. Oldridge [5] estimated that a lack of PA contributes to between 1.5% and 3.0% of direct healthcare costs in developed countries. Colman and Walker [6] estimated that in a population of 10 million people, where half of the population is too inactive to enjoy health benefits from PA, the costs of insufficient PA can be up to €910 million a year [6]. The loss of health due to insufficient PA and its economic impact on society emphasizes the importance of stimulating people to become more physically active, which can result in better public health and thereby reduce health care costs [7–9].

Older people especially face physical disabilities more often, resulting in substantial (perceived) barriers to PA. Moreover, particularly among this group physical inactivity is associated with greater risk of serious health problems [1, 10]. Since the aging population places increasing demands on healthcare budgets [11, 12], effectively stimulating PA with interventions against acceptable costs among older adults is of major relevance. Research has shown that even modest increases in PA produce substantial health benefits and decreased mortality [13], and that improvements in PA result in savings in health care costs, even within a year [14].

Computer-tailoring is a potentially cost-effective strategy for promoting PA behavior [14–16], as it provides the opportunity to give an individual advice to large populations with minimal costs. Several studies have shown that interventions aimed at personal characteristics of participants and interventions using behavioral change strategies (as applied in most tailoring interventions) are most effective in stimulating PA [15]. As a consequence, the theory- and evidence-based computer-tailored Active Plus intervention was developed [17, 18]. Tailored advice was delivered to participants on three occasions within a four-month period [17]. The intervention was available in different versions: (1) basic print-delivered, targeting psychosocial determinants; (2) environmental print-delivered, targeting environmental determinants in addition to the basic intervention; (3) basic Web-based; and (4) environmental Web-based, with identical content as the environmental print-delivered version [18].

Since there is a high need for cost-effective interventions to reduce healthcare costs within the aging population, the main research question for the current study is: ‘What are the long-term health outcomes and cost-effectiveness of the different versions of the computer-tailored physical activity Active Plus intervention among people aged over fifty?’. Whereas a previous study provided insight into the efficacy of the intervention on PA one year after the intervention started [16], for the current study modelling analyses were performed to provide insight into the intervention’s health effects, which often become more visible when follow-up time increases, mostly beyond the trial period. Using the Chronic Disease Model [19], effects on PA were extrapolated beyond the trial’s time horizon, providing insight into reduction in disease incidences and consequent effects on health-related quality of life and mortality over time, as a result of increased PA. Combining the model outcomes with intervention cost estimates, this study also provides insight into long-term cost-effectiveness of the intervention expressed in costs per quality adjusted life year (QALY). The measure of costs per QALY gained (instead of only effects on PA) enables policy makers to prioritize between different interventions (e.g. comparing cost-effectiveness of a quit-smoking intervention versus a PA intervention).

To our knowledge no other studies have been performed on long-term (i.e. lifetime) cost-effectiveness of computer-tailored PA interventions among older adults. As tailored intervention have the potential to be cost-effective, and reach large populations against minimal cost, even among older adults [20], more insight in its long-term cost-effectiveness is highly needed. Proving the long-term cost-effectiveness of computer-tailored interventions to stimulate PA among older adults, might stimulate the large-scale implementation of these interventions, and thereby prevent the loss of health and lower the impact of the aging population on health care costs.

## Methods

### Design

Four different intervention-versions and a waiting-list control group (who did not receive the intervention until the study was ended) were studied using a clustered RCT with evaluation assessments at the start, after three, six and 12 months. Results on self-reported PA after 12 months were extrapolated to long-term outcomes (i.e. 5-year, 10-year, and lifetime horizon) using the Chronic Disease Model. The current study was registered at the Dutch Trial Register (NTR2297) and approved by the Medical Ethics Committee of Atrium–Orbis–Zuyd (MEC10-N-36).

### Participants and procedures

Participants were recruited via direct mailing in communities of the Municipal Health Council (MHC) regions participating in this study (2010-2011) [21]. Communities were matched on their urbanity, percentage of people with a low SES, percentage of people with a high SES, percentage of immigrants, and the percentage of people aged over 50. Each MHC provided a random sample of eligible participants. Using a random number generator, regions were randomly assigned to one of five research arms. Participants had to be aged over 50 (no maximum age), and needed a sufficient understanding of the Dutch language. No other in- or exclusion criteria were set.

**Figure 1:**
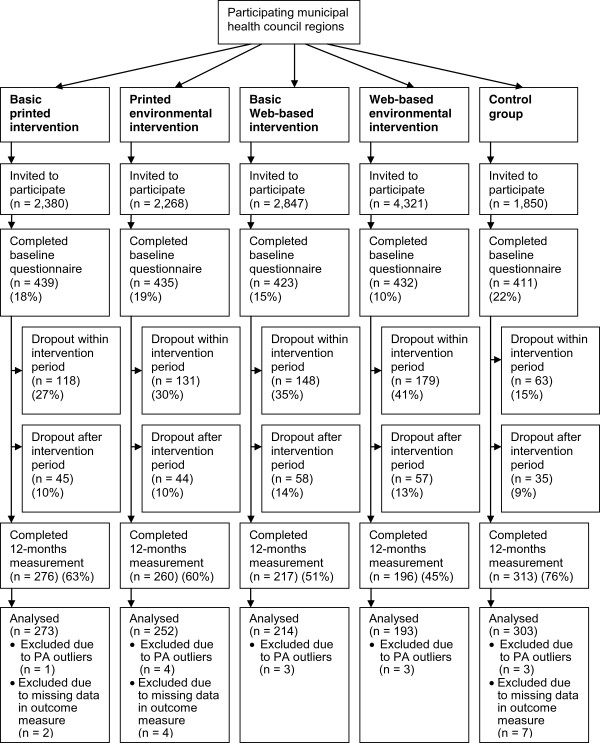
**Flow diagram of the enrolment and dropout of participants during RCT.**

A power calculation (effect size = 0.4, power = 80%, intracluster-correlation coefficient = 0 · 1) showed that at baseline 420 participants were needed per intervention condition (considering a dropout rate of 40% during the 1-year follow-up based on a previous Active Plus study) [21]. Figure 1 provides an overview of the participant numbers included at baseline and 12-months assessment. To reach an equal number of participants per intervention condition, a higher number of invitations had to be distributed in the online intervention regions. Participants provided informed consent before enrolment.

### The Chronic Disease Model

The Chronic Disease Model is a population-based, Markov-type state-transition model that forecasts the development over time in demographics, prevalence of risk factors, disease incidences, and mortality [19]. Markov-type state-transition models are often used to estimate the health effects of PA behaviour [22–24]. The Chronic Disease Model describes the changes in physical (in)activity over time (e.g. the model projects a decrease in PA behavior (i.e. an increased risk of less PA) over time as a result of an increase in age) and the resulting changes of morbidity and mortality for several PA-related chronic diseases (i.e. diabetes, colon cancer, breast cancer, acute myocardial infarctions, stroke) in the Dutch population. The Chronic Disease Model has been described extensively by Hoogenveen and colleagues [19]. The model is based on data provided by national studies with regard to the risk factor distribution and transition, and on data from regional and national registries and registries in general practices with regard to disease incidence and prevalence [19].

Leisure-time PA of the Dutch population in the model’s base year (2007) is described as a continuous distribution expressed in MET-hours. MET (Metabolic Equivalents) presents the energy costs of PA as a multiple of resting metabolic rate. MET-hours thereby include both duration and intensity of PA, and are assumed to have a dose-response relation to PA-related disease incidences. The dose-response regression parameters were obtained by meta-regression on several studies (see Additional file 1). Since literature only provides sufficient evidence on the relation between PA-related diseases and leisure-time PA, work-related PA was excluded from the model.

### Tailored intervention

Active Plus is a computer-tailored, theory- and evidence-based intervention to stimulate or maintain PA in people aged over fifty by targeting pre-motivational constructs (i.e. awareness, knowledge), motivational constructs (i.e. attitude, self-efficacy, social influence, intrinsic motivation, and intention) and post-motivational constructs (i.e. strategic planning, action planning and coping planning) [17]. The intervention was developed using the Intervention Mapping protocol [25], and based on several theoretical models such as the I-Change Model [26], transtheoretical model [27], the health action process approach [28], the precaution adoption model [29], the self-regulation theory [30], and the self-determination theory [31]. Whereas several models and studies have emphasized the importance the participants’ perceptions of their environment in explaining PA behaviour [32–39], additional environmental information was added to the intervention.

In a previous phase of the Active Plus project (2005–2009), the printed intervention was proven to be effective in stimulating physical activity among the over-fifties until one year after the intervention started [40, 41]. In a follow-up study (started in 2010), the intervention was adapted based on the results of the evaluation of the original (print-delivered) intervention, translated into a Web-based version [18], and implemented in an RCT in other regions than the previous Active Plus intervention. The intervention was thus available in a printed and in a Web-based delivery mode, with and without additional environmental information, resulting in the afore mentioned four intervention conditions. Intervention participants received three times tailored advice based on the answers given in previous assessments [17, 18]: (1) after the baseline assessment; (2) two months after the baseline assessment; and (3) within four months after baseline assessment, based on a combination of the first and the second assessment. The specific content of the basic tailored advice depended on the participants’ personal and psychosocial characteristics, PA behaviour, and the extent to which they were planning to change their behaviour [17]. Participants received among others a graphic that provided insight in their own PA behaviour compared to the average PA behaviour of similar others (same age and gender), model stories of similar others, information on the consequences of physical inactivity specifically for their own age group and gender, and suggestions on how to deal with their perceived barriers for physical activity. The intervention with additional environmental components contained the same information as the basic tailored intervention, with additional tailored advice on local PA possibilities and initiatives [42]. Participants received walking and cycling routes tailored to their own neighbourhood, home exercises tailored to their gender, and information on sports opportunities tailored to the opportunities in the participants own neighbourhood, the presence of a chronic physical limitation, and to the participants preferences (e.g. costs, inside or outside, being physically active with others or alone).

The content of the Web-based intervention was identical to the content of the printed intervention; however, the possibility to use interactive components was optimally used (i.e. static modelling pictures and PA exercises were transferred into videos; the printed neighbourhood map was transferred into a Google neighbourhood map; several hyperlinks and a forum were added to the webpage). One day after receiving the online advice, participants in the Web-based intervention received an email with a copy (pdf format) of their advice, enabling them to print and save their tailored advice. The advice sent by email had the same format as the printed version. The tailored advice contained between five and 11 pages of text and illustrations, depending on (changes in) PA behaviour and determinant scores. The tailored advice texts, in the printed letter or on the website, formed the basis of the Active Plus intervention. Intervention components (mainly expressed in pictures/figures/videos or schema’s) were added to the tailored advice to increase the active participation in the intervention. The specific content of the theoretical methods, practical strategies, and intervention components used in the current intervention have been described extensively elsewhere [42].

### Study outcomes

#### Physical activity

PA was measured using the validated self-reported Dutch Short Questionnaire to Assess Health Enhancing Physical Activity (SQUASH), which has a reasonable reproducibility (*r*_spearman_ = 0.58; 95% CI = 0.36–0.74) and relative validity (*r*_spearman_ = 0.45; 95% CI = 0.17–0.66) for the general adult population [43].

#### Model outcomes

Model outcomes are annual QALYs, disease incidence numbers, and healthcare costs. Chronic Disease Model generates healthcare costs by multiplying disease-prevalence numbers with age and gender-specific cost-data from the Dutch Cost of Illness Study [44, 45] and includes future savings in healthcare consumption due to increased PA as well as costs resulting from an increase in life years as a result of more PA [46].

### Data analyses

#### Scenarios applied to the Chronic Disease Model

The Chronic Disease Model was implemented in R [47]. Long-term outcomes were simulated for five scenarios. The first scenario considered care-as-usual (i.e. the reference scenario, receiving no intervention) and was based on the model input data. The remaining scenarios reflected large-scale implementation of different intervention conditions, i.e. offering the intervention once to the entire Dutch population aged over 50 (i.e. 6.2 million people). To increase power and to provide the most reliable estimates of the intervention effect on self-reported PA, scenarios were not defined as single arms of the trial, but presented the printed and Web-based version (combining basic and environmental) as well as the basic and environmental version (combining printed and Web-based).

The observed participation rates in the Active Plus RCT (i.e. 19% for the printed intervention; 12% for the Web-based intervention) [21] are assumed to increase in a real-life setting without additional questions for research purposes. A shortened questionnaire length presumably results in increased response rates for printed questionnaires with an odds ratio (OR) of 1.48 [95% CI = 1.06-2.07] [48] and for Web-based questionnaires with an OR of 1.73 [95% CI = 1.40-2.13] [49]. For the model analyses, the response rates were therefore estimated to be 28% (1.48*19%) for the printed intervention and 21% (1.73*12%) for the Web-based intervention. Finally, since the behaviour can considered to be a habit after one year, and based on the results of another PA intervention in an older population, it was assumed that about 72% of the intervention effects on PA observed at 12 months persisted after these 12 months when the intervention is not continued [50].

#### Baseline differences

ANOVAs (with Tukey’s post hoc-tests) and Chi-square tests were conducted to assess baseline differences in demographics and PA between the research arms. Following guidelines of the SQUASH [43], 14 respondents were excluded because of PA levels of more than 6,720 minutes per week since being physically active for 7 days per week, over 16 hours per day was considered to be unreliable.

Hierarchical logistic regression analyses were performed to study whether any of the demographice characteristics were predictors of dropout at the 12 month measurement, and whether these predictors differed between the research conditions.

#### Intervention effects on physical activity

To calculate the intervention effect, it is assumed that the effect on PA in non-responding participants at 12-months was equal to the control group (in similar age and gender subgroups). This assumption is more conservative than applying multiple imputations, since multiple imputations might overestimate intervention effects [51]. Differences between baseline and 12-months PA were calculated per intervention condition using a paired sample t-test in SPSS. Intervention scenarios combined two intervention groups and hence average (weighted) effects on PA were implemented in the Chronic Disease Model. To correct for baseline differences in demographics, the data on PA was implemented in the Chronic Disease Model per age subgroup (50-64 years, >64 years) and gender subgroup.

The effects of the different intervention conditions on PA were compared to the control group using a linear regression model with PA at 12 months as the dependent variable, and the dummies of the intervention conditions (with the control group as a reference case) as independent variables, and corrected for age, gender, and baseline PA.

Since the current study relies on a clustered randomisation, it can be expected that participants can not be considered totally independently, because participants living within one cluster are more likely to be similar than participants selected at random [52]. However, previous multilevel analyses (including the individual participants and their neighbourhoods as separate levels) have shown that no cluster effects were found. For the current study, participants are therefore considered as independently.

#### Intervention costs

Material and time costs for recruitment and implementation were registered during the RCT. Based on these costs, intervention costs for a real-life setting (using shorter questionnaires, higher response rates and lower printing and postage costs) were estimated (Table 1). More detailed information on the intervention costs is published elsewhere [53]. Costs were divided into fixed costs, that is, costs unrelated to the number of participants (e.g. hosting costs for tailoring software) and variable costs, that is, costs related to the number of participants (e.g. postage and printing costs). Intervention scenarios combined two intervention groups and hence average costs were applied.

**Table 1:** **Fixed and variable intervention costs in a real-life setting per intervention condition (price level 2011)**

	Fixed costs (€)	Variable costs per participant (€)
Printed basic	3300	18
Printed environment	4270	21
Web-based basic	3420	8
Web-based environment	4880	8
Control group	–	–

Note: for more details see the article by Golsteijn et al. [53].

#### Calculation of cost-effectiveness

Incremental cost-effectiveness ratios (ICERs) were calculated by dividing incremental costs (euros) by the health benefits (QALYs gained) resulting from an increase in PA. Economic consequences are presented as: (1) intervention costs per QALY; and (2) total costs per QALY (including healthcare costs being saved as a result of increased PA and increased costs as a consequence of increased life expectancy). Future costs were discounted at 4% and effects on QALYs were discounted at 1.5% [54].

Although there is no official threshold in The Netherlands for the maximum Willingness-to-Pay (WTP), we used a threshold of €20,000/QALY, as this is commonly used for Dutch preventive interventions [55]. When an intervention results in higher effects and lower costs than the comparator, or higher effects and higher costs with an ICER lower than the WTP, then the intervention is considered as cost-effective.

#### Uncertainty analyses

Uncertainty analyses were performed to investigate the robustness of the ICERs. Effectiveness levels on PA (and consequently the effects on QALYs and healthcare costs) were varied using the 95%CI of the RCT in Monte Carlo simulations (100 runs). Using R-package BCEA [56], the results were visualized in a cost-effectiveness plane and a cost-effectiveness acceptability curve (CEAC) representing the probability that one intervention scenario is more cost-effective than the others for a range of WTPs.

Sensitivity analyses were performed regarding the results on a lifetime horizon by applying discount rates of 4% and 0% on both costs and QALYs [54]. Also the response rates for actual implementation (and consequently the intervention costs per participant, since the fixed cost have to be divided by the number of participants) were varied in a sensitivity analysis, taking into account the uncertainty around the measured response rates and the 95%CI of the ratios used to correct the trial based estimate [48, 49].

## Results

### Baseline characteristics

Baseline characteristics of the study population (*N* = 2,140) are shown in Table 2. Participants in the control group and the printed environmental intervention were significantly older than participants in the Web-based basic (*p* = .001; *p* = .002) and the Web-based environmental intervention (*p* < .001; *p* < .001); printed basic intervention participants were significantly older than Web-based environmental participants (*p* = .001). After 12 months, outcome measures were available for 1,235 participants (58%; Figure 1).

**Table 2:** **Baseline characteristics of the participants, mean and standard deviation (SD)**

	Control group (n = 411)	Printed basic (n = 439)	Printed environ. (n = 435)	Web-based basic (n = 423)	Web-based environ. (n = 432)	***P***
Mean age (years) (±SD)	64.2 (±9.5)	63.1 (±8.7)	64.0 (±9.4)	61.8 (±7.1)	60.8 (±7.5)	**.00**
Gender (% men)	49.9	45.9	45.3	52.3	51.3	.22
Education (% low)	50.3	43.5	47.3	46.1	47.8	.40
Paid job (%)	42.8	40.1	43.9	36.8	40.2	.27
MET-hours PA/week (±SD)	45.4 (±40.0)	41.6 (±37.7)	41.5 (±32.1)	42.9 (±38.9)	43.0 (±40.7)	.58

Note: bold numbers reflect a significant difference between the intervention conditions of *p*<.05.

Dropout analyses showed that participants who received additional environmental information (*B* = .398, *p* < .001), participants in Web-based conditions (*B* = .614, *p* < .001), and younger participants (*B* = −.016, *p* = .006) were more likely to dropout from the 12 month follow-up measurement. Demographic predictors of dropout did not differ between the intervention conditions.

### Intervention effects on physical activity

Effects of the intervention conditions on PA after 12 months are presented in Table 3 for the complete case analyses and the analyses using imputed values for participants who dropped out. Only the results using imputation are implemented in the Chronic Disease Model.

**Table 3:** **Overview of the weekly MET-hours spent on leisure PA per intervention condition**

	Baseline		12 months(complete cases)		12 months(imputed)		∆ MET-hours	
	***N***	MET-hours[95% CI]	***N***	MET-hours[95% CI]	***N***	MET-hours[95% CI]	MET-hours[95% CI]	***P***-value
Printed basic	429	41.60[38.02-45.18]	273	47.97[43.74-52.29]	427	44.87[41.08-48.66]	3.06[0.38-5.75]	**.025**
Printed environment	419	41.45[38.37-44.45]	252	50.00[44.91-55.10]	417	44.58[40.87-48.30]	2.94[0.26-5.62]	**.031**
Web-based basic	421	42.89[39.16-46.61]	214	48.57[42.96-54.19]	418	43.71[39.77-47.65]	0.73[-1.94-3.41]	.591
Web-based environment	425	42.95[39.07-46.84]	193	43.16[37.45-48.43]	425	42.74[39.07-46.40]	-0.22[-3.19-2.76]	.887
Control group	402	45.39[41.47-49.30]	303	43.49[39.54-47.44]	397	42.85[39.14-46.55]	-2.20[-5.10-.71]	.139

Note: Differences in MET-hours between both measurements were calculated using the data including imputations for participants who dropped out; bold numbers reflect a significant difference between the intervention conditions of *p*<.05.

Analyses on the data including imputations showed that the printed basic (∆MET-hours = 3.06; 95% CI = 0.38-5.75; *p* = .025) and printed environmental intervention (∆MET-hours = 2.94; 95% CI = 0.26-5.62; *p* = .031) resulted in a significant increase in MET-hours. Since the control group decrease PA, the effect of the printed basic intervention (*p* = .022) and the printed environmental intervention (*p* = .030) were even more significant when compared to the control group. The increase in MET-hours of both Web-based interventions was not statistically significant.

### Long-term health benefits

The printed and the Web-based intervention scenarios decreased incidences for PA-related diseases (Table 4). This indicates that although the effect of the Web-based intervention was not statistically significant, it can be clinically relevant. The effects on incidence numbers reduce at longer follow-up times. The printed scenario results in a larger disease incidence decrease than the Web-based scenario, since it was more effective and showed lower dropout rates [16]. Providing additional environmental information resulted in increased incidences compared to the basic intervention, since the effectiveness was lower and dropout was higher.

**Table 4:** **Relative change in incidence numbers for PA-related diseases after extrapolating the Active Plus intervention effects**

	Printed intervention vs. control			Web-based intervention vs. control			Environmental vs. basic		
	5 year	10 year	Life time	5 year	10 year	Life time	5 year	10 year	Life time
Diabetes	-3.1%	-2.8%	-2.0%	-1.3%	-1.0%	-0.6%	1.2%	1.1%	0.8%
Colon cancer	-2.1%	-2.0%	-1.3%	-1.0%	-0.9%	-0.4%	0.8%	0.8%	0.5%
Breast cancer	-0.6%	-0.6%	-0.3%	-0.3%	-0.2%	-0.1%	-0.3%	-0.3%	-0.2%
AMI	-2.3%	-2.2%	-1.4%	-1.1%	-1.0%	-0.4%	1.3%	1.2%	0.8%
Stroke	-1.7%	-1.5%	-0.8%	-0.8%	-0.7%	-0.3%	0.5%	0.5%	0.3%

AMI = Acute myocardial infarction.

**Figure 2:**
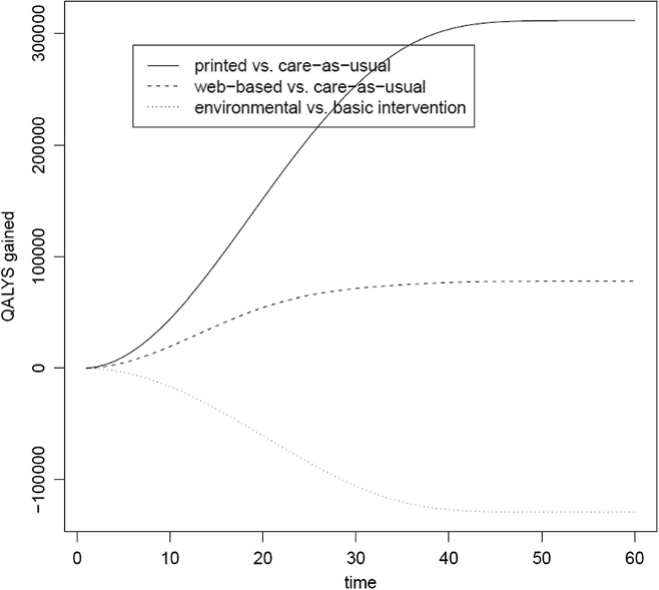
**Delta (absolute) cumulative QALYs gained presented for the printed and the Web-based intervention in contrast to care-as-usual, and for the environmental intervention in contrast to the basic intervention.**

The printed scenario results in more QALYs gained (i.e. 312,000 QALYs on a lifetime horizon) than the Web-based scenario (i.e. 78,000 QALYs on a lifetime horizon). Compared to the basic intervention, the environmental intervention results in a decrease of 129,000 QALYs. These results are visualized in Figure 2, in which the lines present the QALYs gained for the printed and the Web-based intervention in contrast to care-as-usual (i.e. the zero line in the figure), and for the environmental intervention in contrast to the basic intervention (i.e. the zero line in the figure).

### Cost-effectiveness

Considering a WTP of €20,000/QALY, on a 10-year and on a lifetime horizon, both the printed and Web-based scenarios were cost-effective (Table 5). On a 5-year horizon (when health-effects are not that noticeable yet), however, the Web-based scenario was only borderline cost-effective and the printed scenario was not cost-effective.

**Table 5:** **Long-term health effects, costs and cost-effectiveness of the different intervention scenarios**

Time horizon		Web-based intervention	Printed intervention	***Basic intervention***	***Environmental intervention***
**5 years**	*Incremental effects (versus care-as-usual)*				
	QALYs gained	430	1,040	**−**	-330
	*Incremental costs (versus care-as-usual)*_*a*_				
	Intervention costs	10	30	20	20
	Total costs_b_	10	30	**−**	0
	*Incremental intervention costs per QALY gained*_*c*_				
	Versus care-as-usual	20,500	29,700	**−**	**−**
	Web-based vs. Printed/ Environment vs. Basic	**−**	36,180	**−**	-6,500
	*Incremental total costs per QALY gained*_*c*_				
	Versus care-as-usual	20,760	27,520	**−**	**−**
	Web-based vs. Printed/ Environment vs. Basic	**−**	32,290	**−**	-8,690
**10 years**	*Incremental effects (versus care-as-usual)*				
	QALYs gained	1,990	5,120	**−**	-1,700
	*Incremental costs (versus care-as-usual)*_*a*_				
	Intervention costs	10	30	20	20
	Total costs_b_	20	40	**−**	0
	*Incremental intervention costs per QALY gained*_*c*_				
	Versus care-as-usual	4,410	6,010	**−**	**−**
	Web-based vs. Printed/ Environment vs. Basic	**−**	7,020	**−**	-1,260
	*Incremental total costs per QALY gained*_*c*_				
	Versus care-as-usual	8,450	7,590	**−**	**−**
	Web-based vs. Printed/ Environment vs. Basic	**−**	7,040	**−**	-470
**Lifetime**	*Incremental effects (versus care-as-usual)*				
	QALYs gained	9,150	44,380	**−**	-16,770
	*Incremental costs (versus care-as-usual)*_*a*_				
	Intervention costs	10	30	20	20
	Total costs_b_	90	330	**−**	-90
	*Incremental intervention costs per QALY gained*_*c*_				
	Versus care-as-usual	960	690	**−**	**−**
	Web-based vs. Printed/ Environment vs. Basic	**−**	620	**−**	-130
	*Incremental total costs per QALY gained*_*c*_				
	Versus care-as-usual	10,100	7,500	**−**	**−**
	Web-based vs. Printed/ Environment vs. Basic	**−**	6,830	**−**	5,290

_a_In millions of euros; _b_Intervention costs corrected for health-care cost; _c_calculated according to the formula ICER = (Costs_i_-Costs_c_)/(Effects_i_-Effects_c_).

Comparing the Web-based to the printed scenario, on a 10-year and on a lifetime horizon monetary savings gained by implementing the Web-based intervention instead of the printed intervention do not outweigh its lower effects (i.e. the ICER of the Web-based intervention versus the printed intervention is lower than the WTP). Since both the cost and the effects are negative when comparing the Web-based intervention to the printed intervention, in this case the WTP can be seen as the minimum amount of money that the society would like to save by implementing a less effective intervention. Since the ICER of the Web-based intervention compared to the printed intervention is lower than €20.000, the monetary savings of implementing the Web-based intervention do not outweigh its lower effects, making the printed intervention the preferred intervention condition on a 10 year or a lifetime horizon. In contrast, on a 5-year horizon, the lower intervention costs of the Web-based intervention when compared to the printed intervention do outweigh the loss in effectiveness over this short time horizon. An ICER of €32,290/QALY indicates that for a decrease of one QALY in effect compared to the printed intervention, society would save €32,290, making the Web-based intervention the preferred intervention condition on a 5-year time horizon.

The environmental scenario results in higher intervention costs and lower effects than the basic scenario. However, total costs of the environmental scenario on lifetime horizon are lower than the basic scenario, because the basic scenario results in increased life years, and thereby increased healthcare costs. These monetary savings of the environmental scenario do not outweigh the loss in health gains (ICER < €20,000/QALY).

### Uncertainty analyses

**Figure 3:**
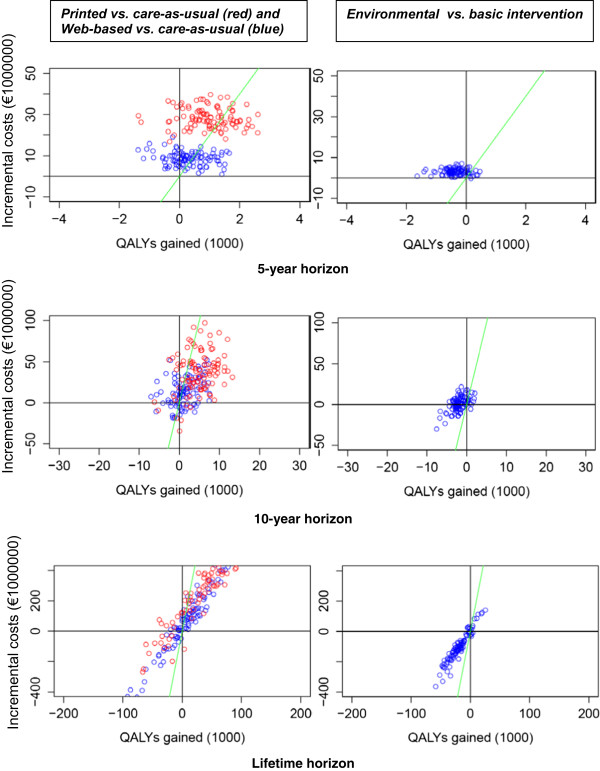
**Cost-effectiveness planes (100 replications) on a 5-year, 10-year and lifetime horizon.**

**Figure 4:**
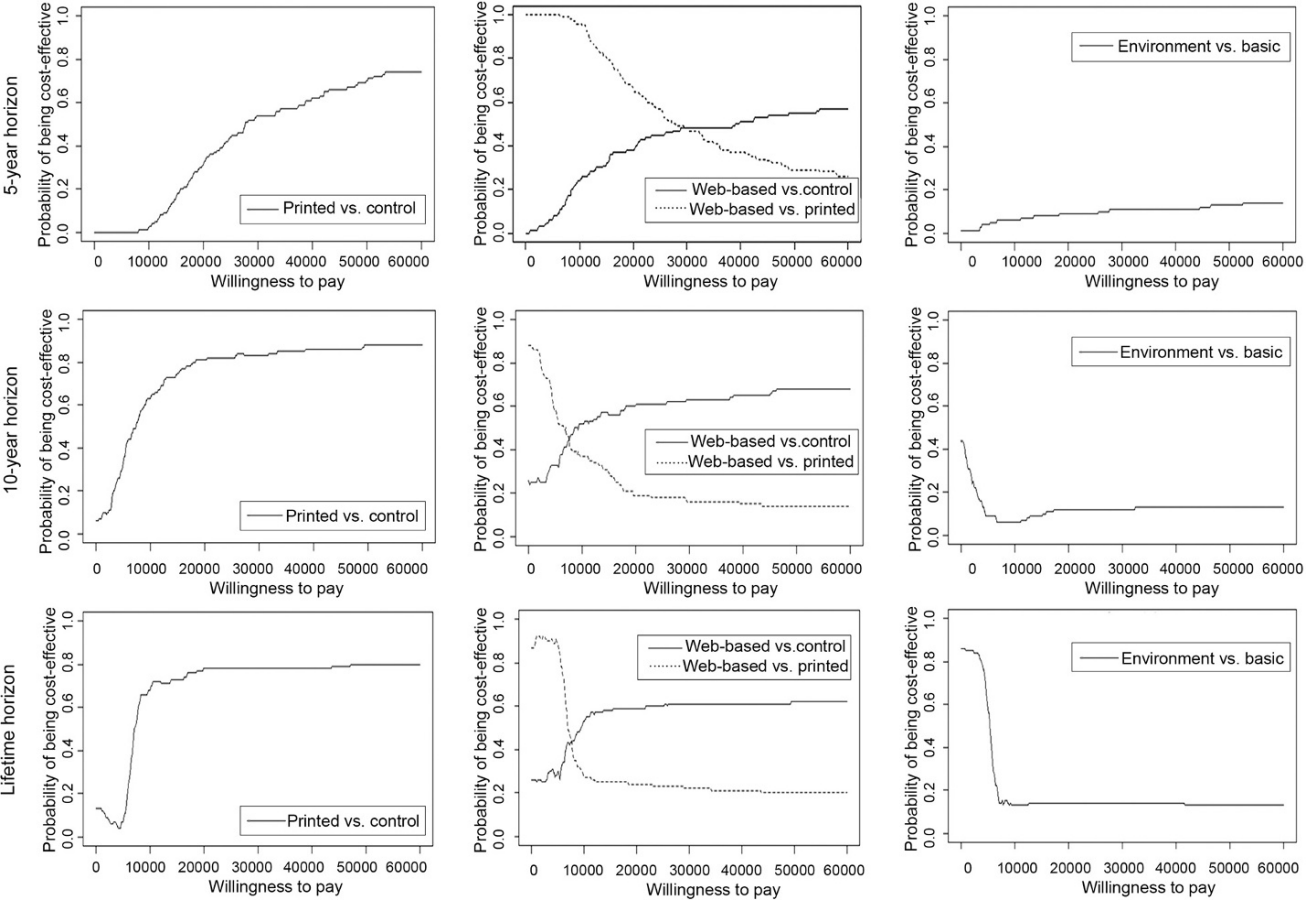
**Cost-effectiveness acceptability curves on a 5-year, 10-year and lifetime horizon.**

Figure 3 shows that on all time horizons, the outcomes of the printed and the Web-based scenarios are mainly located in the north-east quadrant of the cost-effectiveness plane implying that both scenarios are more effective and have higher costs than care-as-usual. On a 5-year horizon, the ICER is mainly located above the WTP (i.e. not cost-effective). On a 10-year and a lifetime horizon, the ICER is mainly below the WTP (i.e. cost-effective). The probability of the printed and Web-based interventions being cost-effective compared to care-as-usual is higher on a 10-year horizon than on a 5-year horizon (Figure 4), since the effects on QALYs and health-care costs become more noticeable over time. On a life-time horizon, probability of being cost-effective slightly declines as a result of a higher life expectancy (due to increased PA), and thereby an increase in healthcare costs.

The outcomes of the environmental scenario (in contrast to the basic scenario) move from the north-west quadrant to the south-west quadrant of the cost-effectiveness plane when the time horizon increases (Figure 3), implying that incremental costs of the environmental scenario decrease with time. At a WTP of €20,000/QALY, the environmental scenario had a probability of less than 20% of being cost-effective on all time horizons (compared to the basic intervention) (Figure 4).

Analyses varying discount and participation rates mainly resulted in comparable ICERs (Table 6). A remarkable difference is observed in the ICER of the Web-based scenario with minimal participation. Since intervention costs of the Web-based intervention mainly exist of fixed costs and less costs per participant when compared to the printed intervention, a lower participation rate results in higher intervention costs per participant than the printed intervention. All other sensitivity analyses resulted in an ICER < 20,000, proving cost-effectiveness of the intervention and confirming the robustness of the results.

**Table 6:** **Uncertainty analyses varying the participation rates and the discount rates of the results on a life time horizon**

Analysis		Web-based intervention	Printed intervention	Environmental intervention vs. basic
**Primary**	Incremental costs per QALY gained_c,d_			
	Versus care-as-usual	10,100	7,500	**−**
	Web-based vs. Printed/Environment vs. Basic	**−**	6,830	5,290
**Minimal participation**_**a**_	Incremental costs per QALY gained_c,d_			
	Versus care-as-usual	20,170	7,480	**−**
	Web-based vs. Printed/Environment vs. Basic	**−**	6,510	5,180
**Maximum participation**_**b**_	Incremental costs per QALY gained_c,d_			
	Versus care-as-usual	9,190	7,450	**−**
	Web-based vs. Printed/Environment vs. Basic	**−**	7,060	4,760
**4% discount on costs and effects**	Incremental costs per QALY gained_c,d_			
	Versus care-as-usual	15,360	12,150	**−**
	Web-based vs. Printed/Environment vs. Basic	***−***	11,340	8,890
**0% discount on costs and effects**	Incremental costs per QALY gained _c,d_			
	Versus care-as-usual	16,580	13,800	**−**
	Web-based vs. Printed/Environment vs. Basic	**−**	13,310	11,100

_a_The lowest boundary of the 95% CI of the OR used to calculate the response rate; _b_The highest boundary of the 95% CI of the OR used to calculate the response rate; _c_In millions of euros; _d_calculated according to the formula ICER = (Costs_i_-Costs_c_)/(Effects_i_-Effects_c_).

## Discussion

The printed and Web-based intervention result in decreased disease incidences and increased QALYs and thus have an important contribution to public health. On a lifetime horizon, the printed and Web-based interventions both have acceptable costs per QALY gained when compared to care-as-usual, and are thus cost-effective. Additional effects of the printed intervention compared to the Web-based intervention are obtained at relatively low additional costs, making the printed intervention the preferred intervention condition in the longer time horizons. Although Web-based tailored interventions have the potential to be more cost-effective [57], this is not confirmed in our study. To further increase cost-effectiveness of Web-based tailored interventions, sustainability of the effects of Web-based interventions should be improved and dropout should be prevented [16].

On a 5-year horizon, however, the cheaper, less effective Web-based intervention is the preferred intervention condition. This implies that when health-related effects are not that noticeable yet, the cheapest intervention is the preferred one. The cost-effectiveness of many prevention interventions is heavily influenced by the time horizon over which analyses are conducted, since they require upfront investments followed by long-term health benefits.

Adding environmental information to the intervention results in higher intervention costs, and did not increase effectiveness as hypothesized [18], making it less cost-effective than the basic intervention. The lack of effect of the extended intervention may be caused by an information overload, resulting in higher dropout from the intervention and decreased effectiveness [16].

To our knowledge, this is the first study on long-term (i.e. lifetime) cost-effectiveness of different delivery modes of computer-tailored PA interventions among older adults. A study by Lewis et al. [58] provided insight into costs associated with internet and print-delivered PA interventions; however, these insights were not presented in relation to the effects of the interventions. A review on the cost-effectiveness of PA interventions showed that behavioural interventions reached recommended PA levels at a cost of 800 euros per participant over a 12-month period [59]. Another review showed that high-intensity, personalized behaviour-change programs was one of the least cost-effective strategies to promote PA, while these also had one of the largest effect sizes [60]. Unfortunately, neither review identified costs per QALY gained, decreasing its comparability with studies on other health behaviours, nor did they include cost-effectiveness over a longer time horizon.

### Strengths and limitations

A main strength of this study is that cost-effectiveness of the intervention is extrapolated beyond the time horizon of the RCT using a model-based approach, resulting in summary measures of population health [19]. However, modelling unavoidably involves making assumptions, which increases the outcomes’ uncertainty. One of the assumptions made was on the persistence of the intervention effect. Based on the results of another PA intervention in an older population, it was assumed that about 72% of the intervention effects on PA observed at 12 months persisted [50]. A study of McAuley et al that was performed among older adults showed a persistence of 85% of the PA behaviour from year 1 (M = 148.80) to year 5 (M = 126.72) after a six month intervention period [61]. A study on the Diabetes Prevention Program, showed a persistence of 76% of the PA behaviour from the end of a lifestyle intervention (where an increase 21.5 met h/week was observed) to year five (were 16.3 met h/week of the PA behaviour was maintained) [62]. Compared to both studies, and taken into account that the Chronic Disease Models additionally projects a decrease in PA behaviour over time as a result of an increase in age, our assumption on the persistence of the intervention effect is relatively conservative.

Other important assumptions made were on the population being reached by the intervention, the effect in participants who dropped-out, and on the immediate dose-response relationship between PA-levels and PA-related disease risks. In reality, a more complex relation might exist with all past PA-levels and other affecting risk behaviours (e.g. smoking). Furthermore, the reach of the intervention might differ due to the use of different recruitment strategies. However, the current approach is in line with estimates from literature, and in most cases assumptions were made as conservative as possible. Sensitivity analyses making variations in assumptions, proved the robustness of the results in this paper.

A disadvantage of the CDM is that it only keeps track of the probability of having a disease, but not includes the probability of having multiple diseases [19]. Another main aspect of the CDM is its Markov type 1 property, which means that conditional on the current demographics and risk factors, future probabilities are estimated independent of the past behaviours [19]. As it can be expected that, especially in an older population, past PA behaviours will determine ones future health states as well, outcomes might be different if past behaviours were included as well.

Another limitation of the current study is that effectiveness estimates were based on self-reported PA. Although self-reports may be less accurate than objective observations, self-administered questionnaires are valid, most commonly used, and most inexpensive method to use in large-scale studies. However, stronger validation of the effects on PA using objective measurements is recommended [63]. Furthermore, the baseline level of PA observed in the current study population was relatively high. This might be caused by the use of self-reported PA questionnaires. Increasing PA behaviour in a highly active population might not make much health differences. However, the relative risk ratios used in the Chronic Disease Model are based on a broad range of PA behaviours to prevent overestimating of the health effects in populations who were already highly active. Promoting PA in less active populations might result in higher health effects and consequently more positive cost-effectiveness ratios.

A final limitation is that the current modelling analyses were only performed from a preventive perspective (i.e. the effects of PA on a decreased risk on diabetes, colon cancer, breast cancer, acute myocardial infarctions, stroke were calculated), ignoring other potential health related effects of increased PA, like improved mobility and independence, improved muscle strength, cognitive functioning, and mental and emotional well-being, and a decreased risk of falling among older adults [64–69]. Furthermore, by only including myocardial infarction and not including other potential heart diseases (due to a lack of valid data) the health effect of the intervention might be underestimated. If all those aspects were taken into account as well, the effect of the intervention on quality of life might have been even higher.

## Conclusion

In conclusion, our study shows that a tailored PA intervention can improve health and quality of life of people aged over 50 against acceptable costs, and thereby has an important public health contribution. The printed basic intervention has the most potential for being cost-effective in adults aged over fifty.

## Electronic supplementary material

Additional file 1: **Application of the Chronic Disease Model including the risk factor physical (in)activity as a continuous variable.** (DOCX 101 KB)
