# Supplementary material for: Long-term health outcomes and cost-effectiveness of a computer-tailored physical activity intervention among people aged over fifty: modelling the results of a randomized controlled trial
Source: BMC Public Health. 2014 Oct 23;14:1099. doi: 10.1186/1471-2458-14-1099 (PMC4221676; doi:10.1186/1471-2458-14-1099)
Supplement: Supplementary file 1 — Additional file 1: Application of the Chronic Disease Model including the risk factor physical (in)activity as a continuous variable. (DOCX 101 KB) [file 12889_2014_7204_MOESM1_ESM.docx]

**Additional file 1.**

**Application of the Chronic Disease Model including the risk factor physical (in)activity as a continuous variable**

To estimate the long-term effects of the Active Plus intervention, the Chronic Disease Model (CDM) of the National Institute for Public Health and the Environment (RIVM) was used. This model has been described extensively by Hoogenveen and colleagues (Hoogenveen et al., 2010). The CDM model is a Markov-type model that describes the effects of epidemiological risk factors (e.g. smoking, physical inactivity) on morbidity and mortality for multiple chronic diseases in the Dutch population. For the current study, the CDM model including only the risk factor physical (in)activity was applied. Originally, the CDM used three classes for physical activity: norm active, medium active and inactive. For the current study, the risk factor physical activity was modelled as a continuous instead of discrete variable.. We defined physical activity in terms of MET-hours based on recreational activities. The meta-analyses performed to adapt the model, and model equations and model parameters used are discussed below.

The CDM model describes the change of the distribution of physical activity in the population over time, and its effects on morbidity and mortality from activity-related chronic diseases.

Model outcomes are projected survival and disease prevalence numbers, which were used to calculate the net present values over the model time horizon of annual quality adjusted life years (QALYs) and health care costs. Lifetime effects on health care costs were calculated based on disease prevalence probabilities combined with age and gender specific cost-data from the Dutch Cost of Illness Study (Van Baal et al, 2005, 2008). Health care costs included future savings in health care consumption due to an increased physical activity level, for instance lower costs of care for cardiovascular diseases (direct health care costs) as well as future higher costs resulting from an increase in life expectancy (indirect health care costs) (Van Baal et al, 2007).

The changes in continuous physical activity levels over time and the resulting changes of discrete health states were modeled following the method of Manton et al., (1988) and Yashin et al (2011). First, mortality probability values depending on the disease states and incidence probability values depending on current physical activity levels were calculated. , These incidence and mortality probability values were used to update distribution over the health states. Next, the distribution of physical activity levels for each health state was updated taking account of the effects of incidene and mortality and the change over time related to aging. The method of Yashin assumes a normal distribution. However, the distribution of physical activity is skewed to the right, and hence the physical activity levels were first log-transformed. The method Yashin assumes quadratic risk functions. Therefore, we transformed the log-linear dose-response risk functions on the original activity values to a quadratic function on the log-transformed values. The coefficients of the log-linear risk function were obtained by meta-regression on a selection of studies presenting dose-effect relations between activity levels and disease incidence.

**Assessment of disease incidence risk functions**

***Meta analyses***

The risk functions describe the relation between continuous physical activity level and disease incidence risks. The physical activity unit we used was MET-hours from recreational activities. Because physical activity is assumed continuous, we can interpret the risk functions as dose-response functions. To assess these risk functions, we used relative risk values from epidemiological studies presented in literature. The selection criteria we used were: cohort study, published since 1995, mainly Caucasian population, recreational activities (i.e. leisure and transport walking and cycling, sports or gardening, excluding domestic chores and other odd jobs performed), activity unit MET-hours including a range of at least 50 MET-hours between the average and the extremes, and adjustment for other lifestyle risk factors. Our search strategy started with a search in PubMed using the keywords ‘physical activity’, ‘cohort’, and name of disease. Then starting with one or more retrieved articles, we used references (to and from) to find more articles. Moreover we used meta-analyses (although they used different selection criteria) to check our study selections. The diseases included in our analyses were those that are considered to be causally related to physical activity (Surgeon General, 1996), i.e. acute myocardial infarction (AMI), stroke (CVA), diabetes mellitus (DME), colon cancer, and breast cancer.

We assumed a log-linear relation between physical activity and the disease incidence. For each disease involved we made the following calculation steps. For each study selected for the meta-regression analyses (see Table 1) we fitted a linear regression function on the log-transformed relative risk values, using the method described by Greenland and Longnecker (1992). The independent values were the calculated mid-points of all activity classes. We compared the calculated relative risk values for each activity class to the reported ones to assess the validity of the log-linear risk functions. For almost all studies the calculated values were located between the reported confidence bounds for all activity classes. We found that the dose-response curves were consistently steeper for the studies with limited activity ranges. However, we were unable to assess a log-quadratic dose-response function. Therefore, we restricted the analyses to all studies where the differences between the mid-points of the extreme activity classes were at least 50 METs. After calculating the study-specific dose-response parameter values, we combined them using a meta-regression model (routine metaphor, Viechtbauer 2010), with the study being the random component. We explored the fixed effect of age, since relative risk values generally decrease with increasing age (see e.g. Durazo-Arvizu, 1997 for BMI, and Malarcher, 2000 for smoking). However, we could not assess a significant effect of age for any of the chronic diseases involved. We therefore assumed the risk function being independent of age.

Table 1: summary of the relations identified between continuous PA (met-hours) and disease incidence risks

|  | **(Sub)population** | **Log (RR)** | **Stand. Error** |
| --- | --- | --- | --- |
| **BREAST CANCER** |  |  |  |
| Maruti et al (2008) | All | 0.0056 | 0.0023 |
| Howard et al (2009) | All | -0.0011 | 8e-04 |
|  | Pre-menopause | -0.0027 | 0.0019 |
| Lee et al (2001) | All | 0.0018 | 0.0029 |
| **Summary** |  | **-0.0019** | **0.00119** |
| **COLON & RECTAL CANCER** |  |  |  |
| Wei et al (2004) | All | -0.0211 | 0.0028 |
| Friedenreich et al (2006) | Colon | -0.0024 | 0.0016 |
|  | Rectum | 0.0032 | 0.0021 |
| Howard et al (2008) | All | -0.0051 | 0.0014 |
| **Summary** |  | **-0.0062** | **0.0051** |
| **DIABETES** |  |  |  |
| Hu et al (2001) | <65 | -0.0155 | 0.0021 |
|  | >=65 | -0.0069 | 0.0017 |
| **Summary** |  | **-0.0111** | **0.0043** |
| **AMI** |  |  |  |
| Wagner et al (2002) | All | -0.0080 | 0.0036 |
| Tanasescu et al (2002) | All | -0.0077 | 0.0014 |
| Hsia et al (2004) | All | -0.0061 | 0.0021 |
| **Summary** |  | **-0.0073** | **0.0011** |
| **STROKE** |  |  |  |
| Huerta et al (2013) | Men | 0.0011 | 0.0027 |
|  | Women | -0.0143 | 0.0049 |
| Hsia et al (2004) |  | -0.004 | 0.0015 |
| **Summary** |  | **-0.0049** | **0.0040** |

Note: RR = Relative risk

***Model equations***

We assumed the MET-hours from recreational activities being log-normally distributed in the population. The mean and variance of the initial distribution were estimated using data from the Active Plus intervention study, stratified by gender and 10-year age-classes. The resulting parameter estimates were smoothed and interpolated, resulting in values for all age-years separately. To describe the change of the distribution of the activity level in a cohort over time we used the drift-diffusion model described by Manton et al. (1988) and Yashin et al. (2011), describing the distribution as a result of three causes: drift, diffusion and mortality. Drift is the fixed 1-year change of physical activity of individuals, diffusion is the random change. Moreover, the distribution changes because inactive individuals have higher mortality risks than active individuals. Manton et al. have shown that under three assumptions the distribution of physical activity is normal over time, i.e. initial normal distribution, linear drift and diffusion, and quadratic mortality risks. Then the change of the distribution over time is fully defined by the change of mean values and variance only. We modelled the mortality risks through the activity-related chronic diseases that were included in the model. That means, inactive individuals have higher disease incidence risks (new cases), and individuals with diseases (prevalent cases) have higher mortality risks. Following the specification of the drift-diffusion model the incidence risks were defined as quadratic functions of the activity level. Note, that in our model we used log-transformed activity levels.

The model equations can be separated in two groups of equations. The first group consists of the equations to calculate all model parameters, including the initial distributions. The second group consists of the equations that describe the 1-year calculation steps of the model. Since all model parameters are specified by age, we use the symbol a (age) in the first group. Because the calculation steps are over time, we use the symbol t (time) in the second group. For any time t, the age value is notated as a(t), where we implicitly assume the initial age value, i.e. at time t=0, given.

**Calculation of model parameters**

***Quadratic disease incidence function***

From the literature search we calculated a log-linear function, that describes the relative incidence risk as a log-linear function of the continuous activity level. In our model we used a quadratic function of the log-transformed activity level. We calculated the parameters of the quadratic function by calculating the 0^th^, 1^st^ and 2^nd^ order derivative values of the log-linear risk function in the mean (log-transformed) activity level.

The relation between the log-linear incidence function and the quadratic function:

$$\exp(\beta\cdot e^{Z})=\exp(\beta\cdot e^{\mu+\delta})=\exp(\beta\cdot e^{\mu}\cdot e^{\delta})=C\cdot g(\delta)$$

with:

Z log-transformed activity level, with mean value µ

β coefficient of log-linear risk function

δ stochastic variable that is defined as: δ = Z - µ

C multiplicative scalar

We calculated the 0^th^, 1^st^ and 2^nd^ order derivative values of function g around mean value µ:

0^th^ order derivative: $g(\delta)=\exp(\beta\cdot e^{\mu}\cdot e^{\delta})$,

with value: $g_{0}=g(0)=\exp(\beta\cdot e^{\mu})$

1^st^ order derivative: $g'(\delta)=\beta\cdot e^{\mu}\cdot e^{\delta}\cdot\exp(\beta\cdot e^{\mu}\cdot e^{\delta})$,

with value: $g_{1}=g'(0)=\beta\cdot e^{\mu}\cdot\exp(\beta\cdot e^{\mu})$

2^nd^ order derivative: $g''(\delta)=\beta\cdot e^{\mu}\cdot e^{\delta}\cdot(1+\beta\cdot e^{\mu}\cdot e^{\delta})\cdot\exp(\beta e^{\cdot\mu}\cdot e^{\delta})$

2^nd^ order derivative value: $g_{2}=g''(0)=\beta\cdot e^{\mu}\cdot(1+\beta\cdot e^{\mu})\cdot\exp(\beta\cdot e^{\mu})$

Because the mean value µ depends on age, we introduce the symbol a here:

$$g(\delta;a)\approx g_{0}(a)+g_{1}(a)\cdot\delta+½\cdot g_{2}(a)\cdot\delta^{2}$$

with g_0_(a) = 1, g_1_(a) = $\beta\cdot e^{\mu(a)}$, and g_2_(a) = $(1+\beta\cdot e^{\mu(a)})\cdot\exp(\beta\cdot e^{\mu(a)})$ respectively. In this way we defined the quadratic disease incidence function up to a multiplicative factor. The value of the multiplicative factor was calculated by calculating the mean value of the disease incidence function, for each age-year a separately:

$$inc_{d}(a)=inc_{d,0}(a)\cdot E_{\delta}(g_{d}(\delta;a))$$

$$\begin{matrix} E_{\delta}(g_{d}(\delta;a))=E_{\delta}(g_{d,0}(a)+g_{d,1}(a)\cdot\delta+½\cdot g_{d,2}(a)\cdot\delta^{2})= \\ g_{d,0}(a)+g_{d,1}(a)\cdot E(\delta)+½\cdot g_{d,2}(a)\cdot E(\delta^{2})=g_{d,0}(a)+½\cdot g_{d,2}(a)\cdot\sigma^{2} \end{matrix}$$

$$inc_{d,0}(a)=\frac{inc_{d}(a)}{g_{d,0}(a)+½\cdot g_{d,2}(a)\cdot\sigma^{2}}$$

with:

a age

σ^2^ variance of activity level Z

inc_d_ known disease d incidence probability

inc_d,0_ calculated baseline disease d incidence probability value

***Quadratic mortality function***

The 1-year all-cause mortality probability depends on the mortality probabilities that uniquely can be attributed to the diseases included in the model, and a rest term related to the other causes of death.

$$m(Z;t)=\sum_{d} P_{d}(Z;t)\cdot am_{d}(a(t))+m_{oc}(a(t))$$

with:

m(Z;t) calculated 1-year all-cause mortality probability on time t

P_d_(Z;t) calculated disease d probability value on time t

am_d_(a) known disease d related attributable mortality at age a (see Van Baal et al., 2010)

m_oc_(a) calculated mortality probability for other causes of death at age a (see below)

We assumed the disease probability function having the same quadratic form as the disease incidence function (see Hoogenveen et al., 2010), up to a multiplicative factor.

$$P_{d}(Z;a)=P_{d,0}(a)\cdot(g_{d,0}(a)+g_{d,1}(a)\cdot\delta+½\cdot g_{d,2}(a)\cdot\delta^{2})$$

$$P_{d,0}(a)=\frac{P_{d}(a)}{g_{d,0}(a)+½\cdot g_{d,2}(a)\cdot\sigma^{2}}$$

with:

P_d_ known disease d probability value

P_d,0_ calculated baseline disease d probability value

In this way, the all-cause mortality probabilities are quadratic functions of the log-transformed activity level. We assumed that the other causes mortality probability does not depend on activity level, thus:

$$m_{oc}(a)=E_{Z}(m(Z;a)-\sum_{d} P_{d}(Z;a)\cdot am_{d}(a))=m(a)-\sum_{d} P_{d}(a)\cdot am_{d}(a)$$

with:

P_d_(a) known disease d probability value at age a

**Calculations for each 1-year model time step**

***1-year change of probability distribution of activity level***

Since we assumed the activity level (after log-transformation) being normally distributed, we can describe the change of the distribution by describing the change of the mean and variance according to Manton et al. [1988]. The individual activity level changes because of fixed changes (drift) and random changes (diffusion). Moreover, the distribution, i.e. conditional on survival, changes because mortality risks depend on the activity level. So, we first define the change of the stochastic individual activity level:

$$Z(t+1)=Z(t)+A_{0}(a(t))+A_{1}(a(t))\cdot Z(t)+dw\cdot\varepsilon$$

The mortality risk function is quadratic in Z (see above):

$$m(Z(t);t)=b_{0}(a(t))+b_{1}(a(t)).Z(t)+½\cdot b_{2}(a(t))\cdot Z(t)^{2}$$

By combining both formulas, the formula of the change of the mean log-transformed activity level is:

$$\mu(t+1)=\mu(t)+A_{0}(a(t))+A_{1}(a(t))\cdot\mu(t)-V(t)\cdot(b_{1}(a(t))+b_{2}(a(t))\cdot\mu(t))$$

Likewise the formula of the change of the variance of the log-transformed activity level is:

$$V(t+1)=V(t)+\Sigma+2\cdot A_{1}(a(t))\cdot V(t)-V(t)\cdot B(a(t))\cdot V(t)$$

with:

t discrete time (years)

Z log-transformed activity level

A_0_, A_1_ parameters of fixed change of log-transformed activity level (drift)

dw scale factor of random change of log-transformed activity level (diffusion)

ε unit 1-year random change (‘white noise’)

µ(t), V(t) mean value and variance of Z(t) respectively

= dw^2^ variance related to random change

We had no longitudinal data to calculate the parameters A_0_, A_1_ and dw. Therefore, we calculated the parameter A_0_ by taking the difference between the mean activity levels for successive age-years that were available from the cross-sectional data. This change estimation can be interpreted as the net activity change over time (Kassteele Jv et al., 2012). We assumed the parameters A_1_ and dw having value 0.

***Current disease incidence probability***

The disease incidence probability value on time t is calculated as:

$$\begin{matrix} inc_{d}(t)=inc_{d,0}(a)\cdot\\ \{g_{d,0}(a(t))+g_{d,1}(a(t))\cdot[E(Z(t)-\mu(a(t)))]+½\cdot g_{d,2}(a(t))\cdot[E(Z(t)-\mu(a(t)))^{2}] \end{matrix}$$

The linear and quadratic terms can be simplified:

$$E(Z(t)-\mu(a(t)))=E(Z(t))-\mu(a(t))$$

$$\begin{matrix} E(Z(t)-\mu(a(t)))^{2}=E(Z(t)-E(Z(t)))^{2}+E((E(Z(t))-\mu(a(t)))^{2}= \\ V(Z(t))+E((E(Z(t))-\mu(a(t)))^{2} \end{matrix}$$

In conclusion, based on the adapted model equations and model parameters, the CDM model is now able to estimate the morbidity and mortality effects of interventions on physical activity modeled as a continuous instead of discrete risk factor.

***References***

Durazo-arvizu R, Mcgee D, Li Z, Cooper R. Establishing the nadir of the body mass index-mortality relationship: a case study. J Am Stat Assoc. 1997 Dec;92(440):1,312-9.

Friedenreich C, Norat T, Steindorf K, Boutron-Ruault MC, Pischon T, Mazuir M, Clavel-Chapelon F, Linseisen J, Boeing H, Bergman M, Johnsen NF, Tjønneland A, Overvad K, Mendez M, Quirós JR, Martinez C, Dorronsoro M, Navarro C, Gurrea AB, Bingham S, Khaw KT, Allen N, Key T, Trichopoulou A, Trichopoulos D, Orfanou N, Krogh V, Palli D, Tumino R, Panico S, Vineis P, Bueno-de-Mesquita HB, Peeters PH, Monninkhof E, Berglund G, Manjer J, Ferrari P, Slimani N, Kaaks R, Riboli E. Physical activity and risk of colon and rectal cancers: the European prospective investigation into cancer and nutrition. Cancer Epidemiol Biomarkers Prev. 2006 Dec;15(12):2398-407.

Greenland S, Longnecker MP. Methods for trend estimation from summarized dose-response data, with applications to meta-analysis. Am J Epidemiol. 1992 Jun 1;135(11):1301-9.

Hoogenveen RT, van Baal PH, Boshuizen HC. Chronic disease projections in heterogeneous ageing populations: approximating multi-state models of joint distributions by modelling marginal distributions. Math Med Biol. 2010 Mar;27(1):1-19

Howard RA, Freedman DM, Park Y, Hollenbeck A, Schatzkin A, Leitzmann MF. Physical activity, sedentary behavior, and the risk of colon and rectal cancer in the NIH-AARP Diet and Health Study. Cancer Causes Control. 2008 Nov;19(9):939-53

Howard RA, Leitzmann MF, Linet MS, Freedman DM. Physical activity and breast cancer risk among pre- and postmenopausal women in the U.S. Radiologic Technologists cohort. Cancer Causes Control. 2009 Apr;20(3):323-33

Hsia J, Aragaki A, Bloch M, LaCroix AZ, Wallace R; WHI Investigators. Predictors of angina pectoris versus myocardial infarction from the Women's Health Initiative Observational Study. Am J Cardiol. 2004 Mar 15;93(6):673-8.

Hu FB, Leitzmann MF, Stampfer MJ, Colditz GA, Willett WC, Rimm EB. Physical activity and television watching in relation to risk for type 2 diabetes mellitus in men. Arch Intern Med. 2001 Jun 25;161(12):1542-8.

Huerta JM, Chirlaque MD, Tormo MJ, Gavrila D, Arriola L, Moreno-Iribas C, Amiano P, Ardanaz E, Barricarte A, Dorronsoro M, Egüés N, Larrañaga N, Molina-Montes E, Quirós JR, Sánchez MJ, González CA, Navarro C. Physical Activity and Risk of Cerebrovascular Disease in the European Prospective Investigation Into Cancer and Nutrition-Spain Study. Stroke 44(1):111-8

Kassteele Jv, Hoogenveen RT, Engelfriet PM, Baal PH, Boshuizen HC. Estimating net transition probabilities from cross-sectional data with application to risk factors in chronic disease modeling. Stat Med. 2012 Mar 15;31(6):533-43

Lee IM, Rexrode KM, Cook NR, Hennekens CH, Burin JE. Physical activity and breast cancer risk: the Women's Health Study (United States). Cancer Causes Control. 2001 Feb;12(2):137-45.

Malarcher AM, Schulman J, Epstein LA, Thun MJ, Mowery P, Pierce B, Escobedo L, Giovino GA. Methodological issues in estimating smoking-attributable mortality in the United States. Am J Epidemiol. 2000 Sep 15;152(6):573-84.

Manton KG, Woodbury MA, Stallard E. Models of the interaction of mortality and the evolution of risk factor distribution: a general stochastic process formulation. Stat Med. 1988 Jan-Feb;7(1-2):239-56.

Maruti SS, Willett WC, Feskanich D, Rosner B, Colditz GA.A prospective study of age-specific physical activity and premenopausal breast cancer. J Natl Cancer Inst. 2008 May 21;100(10):728-37

Surgeon General. U.S. Department of Health and Human Services. Physical Activity and Health: A Report of the Surgeon General. Atlanta, GA: U.S. Department of Health and Human Services, Centers for Disease Control and Prevention, National Center for Chronic Disease Prevention and Health Promotion, 1996.

Tanasescu M, Leitzmann MF, Rimm EB, Willett WC, Stampfer MJ, Hu FB. Exercise type and intensity in relation to coronary heart disease in men. JAMA. 2002 Oct 23-30;288(16):1994-2000.

Van Baal PHM, Feenstra TL, Hoogenveen RT, De Wit GA: Cost effectiveness analysis with the RIVM Chronic Disease Model. Report No 260706002 2005.

Van Baal PHM, Feenstra TL, Hoogenveen R, De Wit GA, Brouwer WBF: Unrelated medical care in life years gained and the cost utility of primary prevention: in search of a 'perfect' cost-utility ratio. Health Econ 2007, 16:421-433.

Van Baal PHM, Polder J, De Wit GA, Hoogenveen R, Feenstra TL, Boshuizen H, Engelfriet P, Brouwer WBF: Lifetime medical costs of obesity: prevention no cure for increasing health expenditure. PLoS medicine 2008, 5:e29.

Van Baal PH, Hoogenveen RT, Engelfriet PM, Boshuizen HC. Indirect estimation of chronic disease excess mortality. Epidemiology. 2010 May;21(3):425-6.

Wagner A, Simon C, Evans A, Ferrières J, Montaye M, Ducimetière P, Arveiler D. Physical activity and coronary event incidence in Northern Ireland and France: the Prospective Epidemiological Study of Myocardial Infarction (PRIME). Circulation. 2002 May 14;105(19):2247-52.

Wei EK, Giovannucci E, Wu K, Rosner B, Fuchs CS, Willett WC, Colditz GA. Comparison of risk factors for colon and rectal cancer. Int J Cancer. 2004 Jan 20;108(3):433-42.

Wolfgang Viechtbauer (2010). Conducting meta-analyses in R with the metafor package. Journal of Statistical Software, 36(3), 1-48

Yashin AI, Akushevich I, EArbeev KG, Kulminski A, Ukraintseva S: Joint analysis of health histories, physiological state, and survival. Mathematical population studies 2011, 18(4):207-233.
